# Anti-SARS-CoV-2 IgM Secondary Response Was Suppressed by Preexisting Immunity in Vaccinees: A Prospective, Longitudinal Cohort Study over 456 Days

**DOI:** 10.3390/vaccines11010188

**Published:** 2023-01-16

**Authors:** Qiu-Yan Xu, Lin Xie, Xin-Qi Zheng, Xian-Ming Liang, Zhi-Juan Jia, Yan-Yun Liu, Xiao-Yu Liang, Li-Li Liu, Tian-Ci Yang, Li-Rong Lin

**Affiliations:** 1 Centre of Clinical Laboratory, Zhongshan Hospital of Xiamen University, School of Medicine, Xiamen University, Xiamen 361004, China; 2 Institute of Infectious Disease, School of Medicine, Xiamen University, Xiamen 361004, China; 3 Xiamen Boson Biotech Co., Ltd., Xiamen 361021, China

**Keywords:** COVID-19, anti-SARS-CoV-2 IgM, immunity, secondary response, vaccination

## Abstract

To obtain more insight into IgM in anti-SARS-CoV-2 immunity a prospective cohort study was carried out in 32 volunteers to longitudinally profile the kinetics of the anti-SARS-CoV-2 IgM response induced by administration of a three-dose inactivated SARS-CoV-2 vaccine regimen at 19 serial time points over 456 days. The first and second doses were considered primary immunization, while the third dose was considered secondary immunization. IgM antibodies showed a low secondary response that was different from the other three antibodies (neutralizing, total, and IgG antibodies). There were 31.25% (10/32) (95% CI, 14.30–48.20%) of participants who never achieved a positive IgM antibody conversion over 456 days after vaccination. The seropositivity rate of IgM antibodies was 68.75% (22/32) (95% CI, 51.80–85.70%) after primary immunization. Unexpectedly, after secondary immunization the seropositivity response rate was only 9.38% (3/32) (95% CI, 1.30–20.10%), which was much lower than that after primary immunization (*p* = 0.000). Spearman’s correlation analysis indicated a poor correlation of IgM antibodies with the other three antibodies. IgM response in vaccinees was completely different from the response patterns of neutralizing, total, and IgG antibodies following both the primary immunization and the secondary immunization and was suppressed by pre-existing immunity induced by primary immunization.

## 1. Introduction

Coronavirus disease 2019 (COVID-19), caused by the severe acute respiratory syndrome coronavirus 2 (SARS-CoV-2), has turned into a global epidemic and presented tremendous healthcare concerns. According to data reported by the World Health Organization on 07 December 2022, the confirmed cases were 642,379,243, and approximately 6,624,118 cases had died from COVID-19. Currently, the pandemic of COVID-19 is as yet a worldwide challenge as there are continually genetic mutations in the SARS-CoV-2 genome, and variations in its protein S are increasingly reported [1]. For controlling the COVID-19 pandemic, vaccination may be the most efficient strategy. According to data reported by the World Health Organization on 4 December 2022, approximately 12,998,974,878 doses of vaccines for COVID-19 have been inoculated globally (https://covid19.who.int/ (accessed on 4 December 2022). However, the vaccines for COVID-19 have to remain effective against severe COVID-19 infections and deaths caused by COVID-19, including those caused by the delta variant. The massive number of breakthrough infections caused by viral diversification and waning immunity warrants a new vaccine or a third vaccine dose. To combat the ongoing resurgence of the COVID-19 epidemic, the US Food and Drug Administration authorized use of a third dose of booster for all adults after completion of primary vaccination with approved COVID-19 vaccines [2]. This step seems essential because preliminary studies indicated that administration of three doses of the Pfizer–BioNtech mRNA vaccine can neutralize the omicron variant of the virus with an approximately 40-fold decline in the viral titer, but administration of two doses is less effective [3]. Our previous study also indicated that one or more booster vaccine doses are warranted because of waning immunity and a massive number of breakthrough infections caused by viral diversification [4]. A booster dose was shown to be extremely helpful in the fight against COVID-19 and accompanying severe illness and death [5]. According to data from the Centers for Disease Control and Prevention, unvaccinated adults have a nearly six-fold increased risk of testing positive for COVID-19 and a fourteen-fold increased risk of dying from the virus than individuals who had been vaccinated [6]. Therefore, continuous monitoring of the protective effect of COVID-19 vaccines against more virulent variants is critical.

SARS-CoV-2 infections can activate a strong response of B-cells. Within days, the specific antibodies immunoglobulin M (IgM), IgG, and IgA are detectable in patients. At first, the antibodies bind to the virus’ external spike glycoprotein and internal nucleocapsid proteins, then the antibodies block the binding of SARS-CoV-2 to the host cell’s surface receptor angiotensin-converting enzyme 2 (ACE2) effectively [7]. The specific antibody response to SARS-CoV-2 inoculation is still under detailed investigation, and a comparison between protective immunity to SARS-CoV-2 infection in COVID-19 patients and in vaccines is urgently required for guiding decisions from public health employees and guiding information about vaccine management [8]. SARS-CoV-2 antigen-specific antibody response is observed as a measure of protective immunity following the administration of SARS-CoV-2 vaccination [9]. The current vaccines for SARS-CoV-2 induce a robust specific IgG antibody response, which has been the subject of investigation [10]. However, limited data are available on the development and maintenance of vaccine-elicited specific IgM antibody responses. IgM antibodies are generally believed to respond early during viral infections and are expected to neutralize a broader range of viral strains than related IgG antibodies [11,12]. Our previous research demonstrated that the IgM seropositivity rate was only 59.02% two weeks after primary immunization [4]. Notably, it was also reported that a proportion of patients never developed an IgM antibody response [13,14,15]. The reason for the difference in IgM response patterns remains unclear. To obtain more insight into IgM in vaccine-elicited immunity, especially over longer periods of time after three doses of vaccination, we performed a prospective cohort study to longitudinally profile the dynamic response of anti-SARS-CoV-2 IgM antibodies at 19 serial time points over 456 days following serial inactivated CoronaVac vaccination. Additionally, the IgM antibody response patterns in vaccinees after primary immunization and secondary immunization were evaluated. Furthermore, correlation of IgM with three other subsets of anti-SARS-CoV-2 antibodies was analyzed.

## 2. Materials and Methods

### 2.1. Study Design and Participants

The 32 participants from Xiamen Boson Biotech Co., Ltd., Xiamen, China, were the same as those in a previous study from our research team [16]. On 24 January 2021, all participants received the first dose (0.5 mL/dose) of the inactivated CoronaVac vaccine (Sinovac Life Sciences, Beijing, China), the second dose 28 days later (on 21 February 2021), and the third dose 276 d later (on 27 October 2021). In this study, the first and second doses were considered primary immunization, while the third dose was considered secondary immunization. Anti-SARS-CoV-2 IgM antibodies (against the spike protein, IgM), anti-SARS-CoV-2 IgG antibodies (against the spike protein, IgG), anti-SARS-CoV-2 neutralizing antibodies (against the receptor-binding domain (RBD), neutralizing antibody) and anti-RBD total antibodies (against the RBD, total antibody) were serially determined to evaluate responses and durations every 7 d for 28 d following each dose, with 6 more visits (102 d, 132 d, and 248 d after the second dose and 61 d, 92 d, and 180 d after the third dose).

This research (#xmzsyyky2021196) was authorized by the Institutional Ethics Committee of Zhongshan Hospital of Xiamen University, China. This research complied with the Declaration of Helsinki guidelines and national legislation. Written informed consent was provided by all participants.

### 2.2. Laboratory Assays

Around 3 mL of venous blood from all participants who had fasted for no less than 8 h was collected in procoagulant tubes. The blood samples were centrifuged for 10 min with 3000× *g*, and the serum on the upper layer of blood was examined for four subsets of anti-SARS-CoV-2 antibodies within 6 h. The four subsets of anti-SARS-CoV-2 antibodies were examined utilizing the reagent that matched measured with an Autolumo A2000 plus system (Anto Biological Pharmacy Enterprise Co., Ltd., Zhengzhou, China), which employs a chemiluminescence microparticle immunoassay as its basis for functioning. Detection experiments were conducted based on the manufacturer’s instructions and a previous study [4]. The result of the chemiluminescent reaction was evaluated in relative light units (RLU). IgM and IgG antibodies were measured using the S/CO (RLU of samples/cut-off) value, with S/CO ≥1.00 deemed positive and <1.00 deemed negative. A one-step competitive method was used to detected neutralizing antibodies. Specific anti-SARS-CoV-2 neutralizing antibodies in blood samples bound to a horseradish peroxidase-labeled RBD antigen, which then neutralized the binding of ACE2 that had been coated on the microparticles and the RBD antigen. The horseradish peroxidase-labeled RBD antigen not neutralized by specific anti-SARS-CoV-2 neutralizing antibodies formed a complex with ACE2 on the microparticles. The RLU was inversely proportional to the amount of specific anti-SARS-CoV-2 neutralizing antibodies in the sample. The neutralizing antibodies were calibrated, calibration was within range of the First World Health Organization International Standard (NIBSC20/136), and were recorded in international units (IU)/mL [4]. Based on 50% protection from infection with SARS-CoV-2, ≥54.00 IU/mL was regarded as positive, and <54.00 IU/mL was defined as negative [9]. Arbitrary units (AU)/mL were used for total antibody concentration, <8.00 AU/mL was defined as negative, and ≥8.00 AU/mL was defined as positive.

### 2.3. Statistical Analysis

Statistical analyses were conducted using IBM SPSS statistics version 25 (SPSS, Inc., Chicago, IL, USA). After performing the Shapiro–Wilk normality test to assess the normality of distribution, Spearman’s correlation analysis was employed to calculate the correlation coefficient of anti-SARS-CoV-2 antibodies. The *r* values of correlation of the results were categorized as extreme (0.91–1.0), strong (0.71–0.9), moderate (0.41–0.7), weak, or poor (0–0.4). An antibody heatmap was generated using the Pheatmap package with default parameters using R version 3.6.3. The McNemar test was used to compare paired positive conversion. Statistical significance was set at *p* < 0.05.

## 3. Results

### 3.1. Characteristics of Participants

As shown in Table 1, a total of 32 participants who provided blood samples at 19 serial time points over 456 days were included in this study, with 24 women (75%; median age 34 years) and 8 men (25%; median age 36 years). The age distribution was not different between men and women (*p* = 0.287). During the pre- and post-vaccination sampling periods, all participants had no history of COVID-19 infection. All participants were of Han nationality. At the time of vaccination, none of the participants had any of the following symptoms: cough, fever, sore throat, fatigue, diarrhea, runny nose, shortness of breath, muscle aches, or loss of or change in sense of smell and taste. 

### 3.2. Anti-SARS-CoV-2 Antibody Responss to the Vaccines

**Figure 1:**
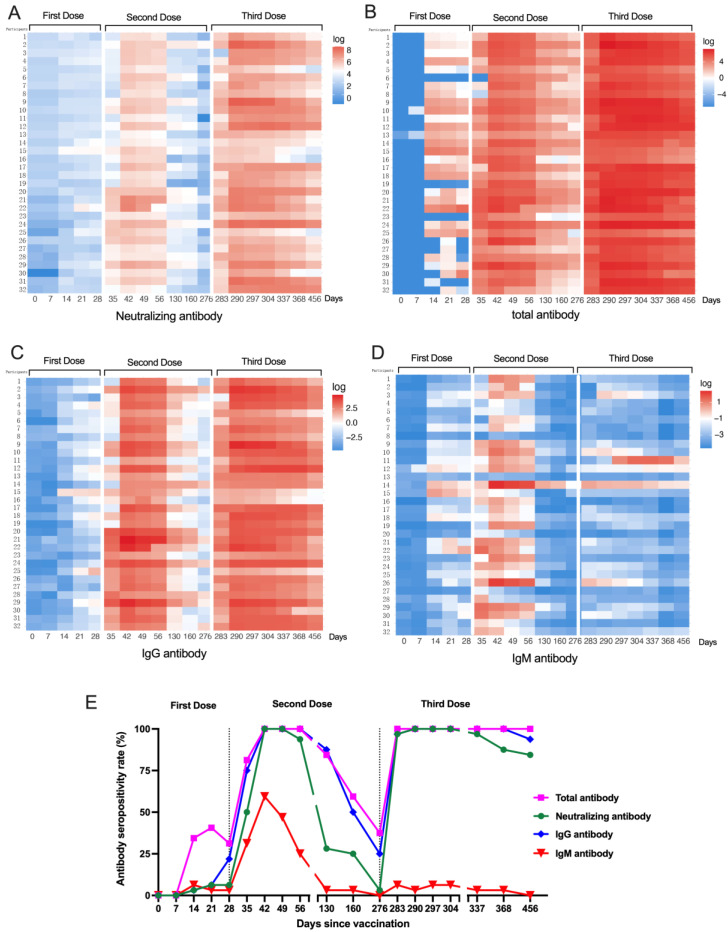
Anti-SARS-CoV-2 antibody response to the vaccines for 32 participants. Heatmaps of the kinetics of anti-SARS-CoV-2 neutralizing antibodies (**A**), anti-SARS-CoV-2 total antibodies (**B**), anti-SARS-CoV-2 IgG antibodies (**C**), and anti-SARS-CoV-2 IgM antibodies (**D**) induced by a three-dose regimen of vaccination. (**E**) Seropositivity rate for the four subsets of anti-SARS-CoV-2 antibodies.

For all thirty-two participants we successfully performed analyses of four subsets of anti-SARS-CoV-2 antibodies (neutralizing, total, IgG, and IgM antibodies) at 19 serial time points within 456 days following vaccination, and then profiled the kinetics of the antibodies. Heatmaps were used to reflect trends in the four subsets of antibodies for individuals based on vaccination dose and time (Figure 1A–D). After the first dose, all four subsets of anti-SARS-CoV-2 antibodies had a minimal response (Figure 1A–D), and the seropositivity rate for all subsets of antibodies was extremely low (Figure 1E). Encouragingly, after receiving the second dose (42 days), neutralizing, total, and IgG antibodies produced a strong response (Figure 1A–D), and the rate of seropositivity significantly increased and reached 100% (32/32). Then, their peaks were maintained for approximately 2 months before they began to decline gradually. In comparison to the second dose of vaccination, the levels of those three antibodies (neutralizing, total, and IgG antibodies) again increased significantly after the third dose and lasted longer, for up to 6 months (Figure 1A–C). In contrast, IgM antibodies increased to a peak of only 59.38% (19/32) and rapidly decreased (Figure 1E) after the second dose (42 days). Unexpectedly, IgM antibodies exhibited only a minimal response of 0.08 (0.03–0.20) S/CO, and the seropositivity rate was only 9.38% (3/32) after the third dose, which was much lower than that after the second dose (Figure 1D,E). The results indicated that IgM antibodies showed a low secondary response, which was different from the other three antibodies, neutralizing, total, and IgG antibodies.

### 3.3. Positive Conversion of Anti-SARS-CoV-2 IgM in the Secondary Immunization

**Figure 2:**
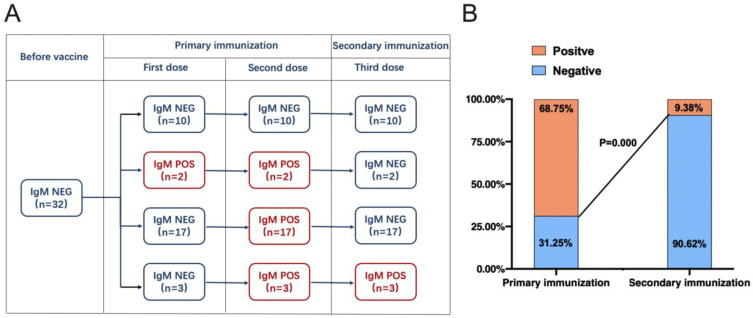
Positive conversion of IgM in primary immunization and secondary immunization. (**A**) Longitudinal positive conversion of anti-SARS-CoV-2 IgM antibodies. (**B**) Positive conversion rates of anti-SARS-CoV-2 IgM antibodies in primary immunization and secondary immunization. The McNemar test was used to compare paired positive conversion between primary immunization and secondary immunization. Abbreviations: NEG: negative, POS: positive.

To better evaluate the response of anti-SARS-CoV-2 IgM antibodies after each of the three doses, positive conversion was longitudinally investigated. There were 10 (31.25% (10/32) (95% CI, 14.30–48.20%)) participants who never achieved positive conversion after vaccination during the three-dose regimen. Their IgM response was minimal after the third dose. Notably, 19 (86.36% (19/22) (95% CI, 70.80–100.00%)) of the participants who had achieved positive conversion after the second dose did not exhibit positive conversion again after the third dose (Figure 2A). According to serial immunization, the seropositivity response rate was 68.75% (22/32) (95% CI, 51.80–85.70%) after primary immunization. Unexpectedly, after secondary immunization, the seropositivity response rate was only 9.38% (3/32) (95% CI, 1.30–20.10%), which was much lower than the seropositivity response rate after primary immunization (*p* = 0.000) (Figure 2B), indicating that the secondary response was suppressed by primary immunization.

### 3.4. Correlation between Anti-SARS-CoV-2 IgM Levels and the Other Three Antibodies

**Figure 3:**
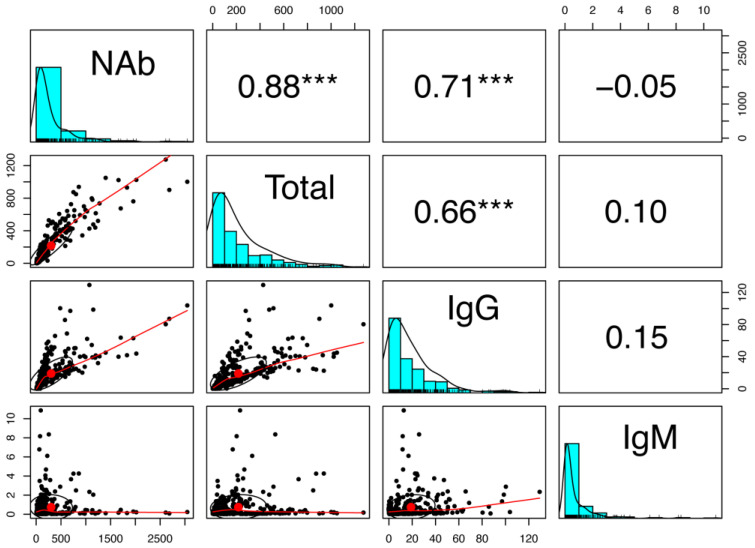
Correlation between anti-SARS-CoV-2 IgM levels and the other three antibodies. Each dot represents the antibodies in one individual at each time point (*n* = 608). ***, significant at the 0.001 probability level. Abbreviations: NAb: anti-SARS-CoV-2 neutralizing antibodies; Total: anti-SARS-CoV-2 total antibodies; IgG: anti-SARS-CoV-2 IgG antibodies; IgM: anti-SARS-CoV-2 IgM antibodies.

The above results indicated that anti-SARS-CoV-2 IgM antibodies showed a low secondary response which was different from the other three antibodies, neutralizing, total, and IgG antibodies. Spearman’s correlation analysis was further conducted to analyze correlations between the four anti-SARS-CoV-2 antibodies. There was a strong correlation between anti-SARS-CoV-2 neutralizing and anti-SARS-CoV-2 total antibodies (r = 0.88; *p* < 0.001), and between anti-SARS-CoV-2 neutralizing and anti-SARS-CoV-2 IgG antibodies (r = 0.71; *p* < 0.001). A moderate correlation was also observed between anti-SARS-CoV-2 total and anti-SARS-CoV-2 IgG antibodies (r = 0.66; *p* < 0.001). However, anti-SARS-CoV-2 IgM antibodies showed a poor correlation with the other three antibodies, neutralizing, total, and IgG antibodies (r = −0.05~0.15; *p* > 0.05). In general, IgM response was different from those of the other three antibodies after prolonged inoculation (Figure 3).

## 4. Discussion

The presence of SARS-CoV-2 changed the way of our lives, having a significant impact on the balance of social life and public health. SARS-CoV-2 is a novel and exceptionally infectious respiratory pathogenic virus that has in practically no time spread across the globe. The pathogenic virus utilizes a protein called spike and its related RBD to interact with ACE2 in host cells. Interaction between viral Spike/RBD and ACE2 on the cell surface is the main fundamental stage in SARS-CoV-2 infections [17]. One of the most effective approaches to significantly lower severe disease and death caused by SARS-CoV-2 infection is vaccination [18]. The program for COVID-19 vaccinations in China was initiated at the end of December 2020, with the vaccine administered in two doses in an interval of 28 days. At the end of September 2021, the National Health Commission of the People’s Republic of China approved the administration of a booster dose of the vaccine at least 6 months after the second dose for persons aged 18 years and older. As per the World Health Organization, immunization is considered a secure, effective, and simple means for protecting the population against the risk of developing severe illness or contracting infectious diseases [19]. One of the principal reasons for administration of the vaccine is utilizing the host immune system to produce specific antibodies for developing resistance against the pathogen, which could be effective against repeated infections or recurrent infections caused by the same pathogen [2]. A crucial parameter in assessing the effects of vaccination and SARS-CoV-2 infection is the level of anti-SARS-CoV-2 antibodies, which facilitates decision-making for subsequent illness prevention and control, as well as vaccine strategy formulation. To date, much research has been performed on neutralizing antibodies, total antibodies, and IgG antibodies after vaccination or natural infection [20,21], and less is known about the characteristics of anti-SARS-CoV-2 IgM antibody response patterns. In this study, we provided a detailed assessment of the kinetics of anti-SARS-CoV-2 IgM during a three-dose schedule of inactivated vaccine administration, with measurements performed at 19 serial time points over 456 days, and detected that anti-SARS-CoV-2 IgM antibodies showed a low response, which was different from the other three antibodies (neutralizing, total, and IgG antibodies). The results were supported by the response of IgM antibodies exhibiting a lower seroconversion peak (59.38% (19/32) after primary immunization and only 9.38% (3/32) following secondary immunization), while the seropositivity rate of neutralizing, total and IgG antibodies peaked at 100% (32/32) after primary immunization or secondary immunization, indicating that IgM antibodies were almost unresponsive after secondary immunization.

Specific antibodies assume a critical part in protective immunity against SARS-CoV-2 infections. A longitudinal understanding of the dynamic changes in antibodies produced by the humoral response after immunization will act as basis for the improvement of successful inoculation and detection strategies. In our study, 32 participants all received three doses of the vaccine and their serum antibodies were successfully measured at 19 serial time points within 456 days following vaccination. The seropositivity rate for four subsets of antibodies was extremely low in the 28 days following receipt of the first vaccine dose. The neutralizing, total, and IgG antibodies produced a strong response, and the rate of seropositivity significantly increased and reached 100% after receiving the second dose, and their peaks were maintained for approximately 2 months before they began to decline gradually. Furthermore, the levels of those three antibodies (neutralizing, total, and IgG antibodies) increased significantly after the third dose and lasted longer, for up to 6 months. The third dose of the vaccine could increase antibody levels which lasted for a longer time [16].

Anti-SARS-CoV-2 neutralizing antibodies against the RBD of the spike protein inhibit the binding of the ACE2 receptor, thereby blocking virus entry into human cells and consequently exerting an antiviral effect. Level neutralization is considered an important predictor of vaccine efficacy [22]. A couple of studies utilized immunoassays to detect neutralizing antibodies for evaluating the values of neutralization in persons who were either COVID-19 patients or vaccinees [23]. The results of neutralizing antibodies were also utilized for evaluating the efficacy of SARS-CoV-2 vaccines [24]. In our study, anti-SARS-CoV-2 total antibodies and anti-SARS-CoV-2 IgG antibodies were both strongly correlated with neutralizing antibodies. For some policies or commercial factors, anti-SARS-CoV-2 total antibodies and anti-SARS-CoV-2 IgG antibodies may be an alternative method for neutralizing antibody detection. Further comparison of the alternation of anti-SARS-CoV-2 total antibodies or anti-SARS-CoV-2 IgG antibodies with neutralizing antibodies is required in future studies. However, we observed that anti-SARS-CoV-2 IgM antibodies showed a poor correlation with the other three antibodies, neutralizing, total, and IgG antibodies. The result also confirmed that IgM response was different from those of the other three antibodies after prolonged inoculation.

The clinical effects of vaccine-induced immunity in protection from infection and severe disease necessitate immediate investigation. To date, detailed monitoring of the adaptive immune response to vaccines can be used as a measure of protective immunity against infection with SARS-CoV-2. IgM antibodies are produced early in the humoral immune reaction against viral infections and provide fast protective immunity. Then, following maturation and isotype class switching, memory IgG antibodies with increased affinity are produced. In our study, 31.25% of the participants never developed IgM during the three-dose vaccination regime. According to serial immunization, the IgM seropositivity rate was 68.75% after primary immunization, 86.36% of positive conversion participants did not exhibit positive conversion again after the third dose, and only 9.38% (3/32) after secondary immunization, which was much lower than that after primary immunization. Moreover, the three individuals with positive IgM after the secondary immunization had already developed positive IgM after the primary immunization. None of the individuals negative after the primary immunization developed IgM antibodies after secondary immunization, and the IgM secondary response to the vaccine was suppressed by pre-existing immunity. A low IgM or negative IgM response has been reported in COVID-19 patients and in vaccines [13,14,15,25]. Alessandra Ruggiero et al. proposed that these noncanonical responses may indicate pre-existing immunity to cross-reactive human coronaviruses [8]. In addition, a significant pairwise correlation was observed among neutralizing, total, and IgG antibodies; however, IgM antibodies showed poor correlation with the other three antibodies. In short, IgM response was different from those of the other three antibodies after prolonged inoculation, which needs further research on the specific mechanism.

To the best of our knowledge, this is the first research to report that the IgM response to COVID-19 vaccines is suppressed by pre-existing immunity. However, the limitations of the study should be considered. Firstly, only 32 uninfected individuals were enrolled, which is a relatively small sample size. Secondly, we only studied IgM developed in response to an inactivated vaccine, without any data about IgM developed after mRNA vaccination. A comparison of the IgM developed in response to an inactivated vaccine with different types of vaccine regimens will be one of our future studies. Thirdly, effective vaccines must elicit a diverse repertoire of antibodies (humoral immunity) and CD8+ T-cell responses (cellular immunity). Unfortunately, the immune cell response was not evaluated in this study. Fourthly, we only performed a prospective cohort study to longitudinally profile the dynamic response of anti-SARS-CoV-2 IgM antibodies following vaccination and detected that the IgM response was different from that of the other three antibodies after prolonged inoculation. It would be better to compare IgM against different pathogens, e.g., IgM response to SARS-CoV-2 and influenza viruses, to confirm the protection by IgM induced by the vaccine. Comparison of IgM against different pathogens for exploring IgM-mediated protective mechanisms would be an interesting aspect that we should focus on in the future. Finally, laboratory data of the participants, such as the level of fasting plasma glucose, glycated hemoglobin(A1c), triglyceride, or cholesterol may be correlated with IgM kinetics, it is a pity that those laboratory data were not evaluated in this study.

## 5. Conclusions

In conclusion, our results indicated that the anti-SARS-CoV-2 IgM response to vaccines was completely different from the response patterns of neutralizing, total, and IgG antibodies following both the primary immunization and the secondary immunization and was suppressed by pre-existing immunity. These findings may contribute to our understanding of the characteristics of anti-SARS-CoV-2 immunity and to the characterization of IgM responses to infection with other pathogens or vaccination.

## Figures and Tables

**Table 1:** Clinical Characteristics of 32 participants to whom three doses of CoronaVac Vaccine were administered.

Characteristics	Value
Gender	
Female, N, (%)	24 (75%)
Male, N, (%)	8 (25%)
Age	
Female age (IQR), years	34 (31–40)
Male age (IQR), years	36 (31–42) ^a^
Race	
Han, N, (%)	32 (100%)
Vaccination schedule	
Days between the first dose and second dose (days)	28
Days between the second dose and third dose (days)	248
SARS-CoV-2 infection	
Before vaccination, N, (%)	0 (0%)
After vaccination, N, (%)	0 (0%)
Presenting COVID-19 symptoms:	
Cough, N, (%)	0 (0%)
Fever, N, (%)	0 (0%)
Sore throat, N, (%)	0 (0%)
Fatigue, N, (%)	0 (0%)
Diarrhea, N, (%)	0 (0%)
Runny nose, N, (%)	0 (0%)
Shortness of breath, N, (%)	0 (0%)
Muscle aches, N, (%)	0 (0%)
Loss of or change to a sense of smell and taste, N, (%)	0 (0%)

^a^*p* = 0.287, compared with female age; N, number; IQR, interquartile range.

## Data Availability

The datasets generated during the current study are available from the corresponding author on reasonable request.
